# Influence of Stimulus Intensity on Multimodal Integration in the Startle Escape System of Goldfish

**DOI:** 10.3389/fncir.2019.00007

**Published:** 2019-02-18

**Authors:** Camille McIntyre, Thomas Preuss

**Affiliations:** Department of Psychology, Hunter College, City University of New York, New York, NY, United States

**Keywords:** multimodal integration, behavioral decision-making, visual loom, inverse effectiveness principle, Mauthner-cell, startle plasticity

## Abstract

Processing of multimodal information is essential for an organism to respond to environmental events. However, how multimodal integration in neurons translates into behavior is far from clear. Here, we investigate integration of biologically relevant visual and auditory information in the goldfish startle escape system in which paired Mauthner-cells (M-cells) initiate the behavior. Sound pips and visual looms as well as multimodal combinations of these stimuli were tested for their effectiveness of evoking the startle response. Results showed that adding a low intensity sound early during a visual loom (low visual effectiveness) produced a supralinear increase in startle responsiveness as compared to an increase expected from a linear summation of the two unimodal stimuli. In contrast, adding a sound pip late during the loom (high visual effectiveness) increased responsiveness consistent with a linear multimodal integration of the two stimuli. Together the results confirm the *Inverse Effectiveness Principle* (IEP) of multimodal integration proposed in other species. Given the well-established role of the M-cell as a multimodal integrator, these results suggest that IEP is computed in individual neurons that initiate vital behavioral decisions.

## Introduction

Integration of sensory information from different modalities is essential for decision-making of appropriately timed behavioral responses. In vertebrates, neurons processing multimodal inputs are found throughout the CNS, prominently the cortical sensory processing areas and superior colliculus in mammals (Meredith et al., 1987; Wallace et al., 1998; Ghazanfar and Schroeder, 2006; King and Walker, 2012), and the optic tectum and hindbrain in birds, amphibians, and fish (Winkowski and Knudsen, 2006; Hiramoto and Cline, 2009; Mu et al., 2012; Medan et al., 2018). Multimodal integration depends on overlapping timing and/or spatial location of unimodal stimuli and typically results in an enhancement of the neural and behavioral response. Specifically, the *Inverse Effectiveness Principle* (IEP) predicts an inverse relationship between individual effectiveness of two unimodal stimuli presented alone and their combined effectiveness, i.e., multimodal integration of two weak stimuli will produce a response that is disproportionately larger than the response evoked by the integration of two strong stimuli. (Meredith and Stein, 1986; Stein et al., 2014). However, establishing causal links between the firing patterns in multimodal neurons and behavioral supporting the IEP has proven difficult (Stanford and Stein, 2007; Holmes, 2009; van Atteveldt et al., 2014). Thus, our goal was to study the IEP phenomenon in a downstream circuit where a distinct behavior can be directly related to sensorimotor neural processing.

We used the startle escape behavior in goldfish, which is controlled by a pair of high-threshold, integrate-and-fire neurons, the Mauthner-cells (M-cells). M-cells receive visual and acoustico-lateralis inputs *via* separate dendrites, and a single action potential (AP) in one M-cell activates contralateral spinal motor circuits for a C-shaped body bend, or “C-start” startle escape response away from a potential threat (Fetcho, 1991; Eaton et al., 2001; Weiss et al., 2006). Importantly, the one-to-one relationship between M-cell threshold and behavioral threshold casually links sensory integration at the M-cell level to startle behavior (Zottoli, 1977; Weiss et al., 2006). Indeed, auditory 8th nerve afferences provide disynaptic (1.8 ms) input *via* mixed electrical and chemical synapses to the lateral M-cell dendrite (Zottoli, 1977; Szabo et al., 2006). Visual information is mediated through a polysynaptic pathway (~20 ms) to the ventral dendrite *via* the optic tectum (Zottoli et al., 1987; Preuss et al., 2006; Dunn et al., 2016). Similarly, abrupt (5 ms) sound pips or gradually increasing (300–1,000 ms) visual looms evoke startles initiated by M-cells (Preuss and Faber, 2003; Preuss et al., 2006; Weiss et al., 2006; Burgess and Granato, 2007; Dunn et al., 2016). Here, we explore the multimodal integration of these two stimuli in goldfish and results indicate supralinear and linear summation of startle rates consistent with the IEP.

## Materials and Methods

### Subjects

Twelve goldfish (*Carassius auratus*) purchased from Billy Bland Fishery (Taylor, AR) of standard body length (mean: 6.15 ± 0.39 cm) and weight (mean: 9.17 ± 1.53 g) maintained in holding tanks (95 L; 30 × 30 × 60 cm; pH 7.2–7.6, 18 ± 1°C) were acclimated for at least 1 week prior to experimentation.

### Apparatus and Stimuli

**Figure 1:**
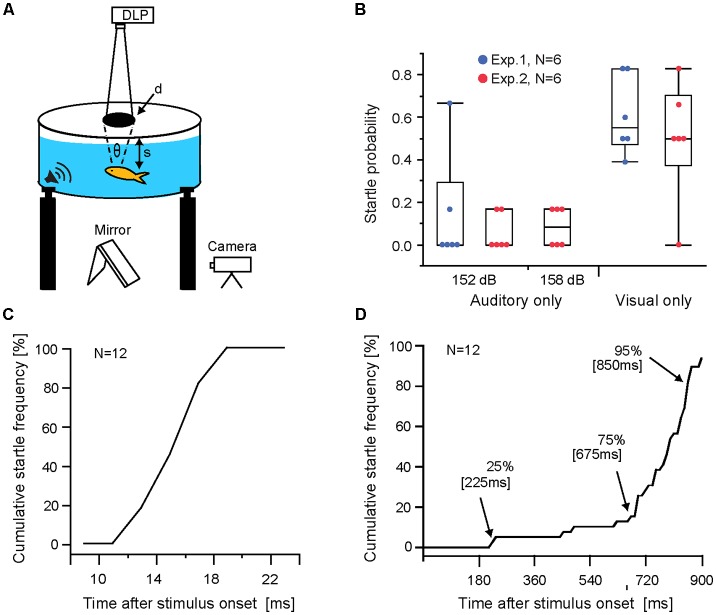
Auditory and visual evoked Mauthner-cell (M-cell) startle responses. **(A)** Schematic of behavioral setup. Visual loom stimuli were projected onto the translucent lid of the experimental tank and sound pips were delivered through underwater loudspeakers (**θ** indicates subtended view angle, d is the diameter of the projected disk, and s is the distance from the lid to the fish). **(B)** Boxplots of startle probabilities to unimodal auditory and visual stimuli used in Exp 1 and 2. Dots indicate data of individual fish. **(C)** Line graph plotting sound evoked cumulative startle frequency vs. response latency for all audio stimuli trials (152 dB and 158 dB re 1 μPa in water). **(D)** Line graph plotting visual evoked cumulative startle frequency vs. response latency. Arrows indicate time points when audio stimuli were triggered in multimodal paradigms. Note: startle escape probabilities increase dynamically during the loom, with most responses occurring between 80% and 95% loom duration.

Experiments were performed in a circular tank (77.5 cm diameter, 30.5 cm deep) located on an anti-vibration table to minimize external mechanosensory cues and covered with a translucent plastic lid, which served as a projection screen for visual stimuli (Preuss et al., 2006). A circular mesh (27.6 cm height; 39 cm diameter) confined the swimming arena. Startle escape behavior was recorded at 1,000 frames/s (Olympus iSpeed2; Figure 1A). Visual loom stimuli consisted of a projected black disc exponentially expanding in size (initial size 8 mm, final size 360 mm, duration 900 ms) produced with custom software (Visloom 1.01) and projected onto the lid with a DLP projector (Plus U4-131; display rate 60 H; Figure 1A). The vertical position of goldfish in the water column varied between 4 and 18 cm resulting in initial view angles subtended on the retina between 2.5 and 11.4 degree (view angle θ = 2* tan^−1^ (d/2 s), where d is the diameter of the projected disk and s the distance from the screen to the fish; Figure 1A). The luminance ratio (L_High_/L_Low_) between background screen (55 lux) and the expanding disc (19 lux) was 1.8.

Auditory stimuli consisted of sound pips (200 Hz; 5 ms; 152 or 158 dB re 1 μPa in water), generated by a stimulator (Master8 AMP), a function generator (Agilent 33210), a power amplifier (Samson Servo 120), and were delivered *via* either of two underwater loudspeakers (Electro-Voice Model UW-30).

### Stimulus Design and Specific Experiments

In goldfish, sound pips produce a sigmoid stimulus response curve (Neumeister et al., 2008), whereas startle rates during a visual loom increase exponentially, i.e., few responses early and peak response rates at 70%–90% of loom duration (Preuss et al., 2006). Accordingly, to produce multimodal stimuli with varying effectiveness, we applied low effective sound pips at different times during a visual loom. However, true stimulus effectiveness can only be assessed after data analysis and revealed that experiment 1 did not include a highly effective stimulus combination. Thus, we performed a follow-up experiment (Exp. 2) in a new set of fish where multimodal stimulus effectiveness was increased by triggering sound stimuli later in the loom and using a higher intensity sound. Stimulus presentation was randomized for every fish.

Experiment 1 was run on six fish, each subjected to four different paradigms, with six presentations for every stimulus namely, audio only (152 dB), visual only, as well as a combination of the two where the audio stimulus was triggered either at 221 or 672 ms after loom onset referred to as AV_Low_ and AV_Med_, respectively.

Experiment 2 (six fish; five stimulus paradigms; six trials each paradigm) included the auditory and visual stimuli of Exp. 1, an added auditory stimulus of higher intensity (158 dB re 1 μPa in water), and two multimodal paradigms where the two auditory stimuli were triggered 832 ms after loom onset (AV_High_152 dB and AV_High_158 dB).

All procedures were performed according to and approved by the Institutional Animal Care and Use Committee (IACUC) of Hunter College^1^.

### Analysis

The predicted linear multimodal summation of startle probability was calculated based on probability observed in visual only and auditory only stimulus trials using the Addition Rule of Probabilities of independent events P(X OR Y) = P(X) + P(Y) − P(X)*P(Y) (Samuels et al., 2012). Mean ± standard deviations (SD) are reported in the text.

## Results

Auditory stimuli evoked overall low response probabilities (Exp. 1, 152 dB, *M* = 0.14 ± 0.26 and Exp. 2, 152 dB *M* = 0.06 ± 0.09; 159 dB *M* = 0.08 ± 0.09; Figure 1B). No significant differences were found between Exp. 1 and 2 for the 152 dB stimulus (*N* = 6; *p* = 0.85 Wilcoxon rank-sum test; Cohen’s *d* = 0.46), or between the 152 dB and 158 dB auditory stimuli in Exp. 2 (*N* = 6; *p* = 0.68; Friedman repeated measure; Cohen’s *d* = 0.35). Essentially, all auditory stimuli showed low effectiveness. In contrast, visual looms elicited sizable mean startle probabilities (Exp. 1, *M* = 0.608 ± 0.18 and Exp. 2, *M* = 0.49 ± 0.27) with no significant difference between Exp. 1 and 2 (*N* = 6; *p* = 0.78, Wilcoxon rank-sum test; Cohen’s *d* = 0.47; Figure 1B).

To illustrate the range of response latencies evoked by auditory stimuli and visual stimuli we combined all responses for a given modality showing that auditory evoked startles occur within a narrow range of latencies (Figure 1C). In contrast, startles in response to visual looms show a wider latency range with most responses occurring between 75%–95% of loom duration (Figure 1D).

**Figure 2:**
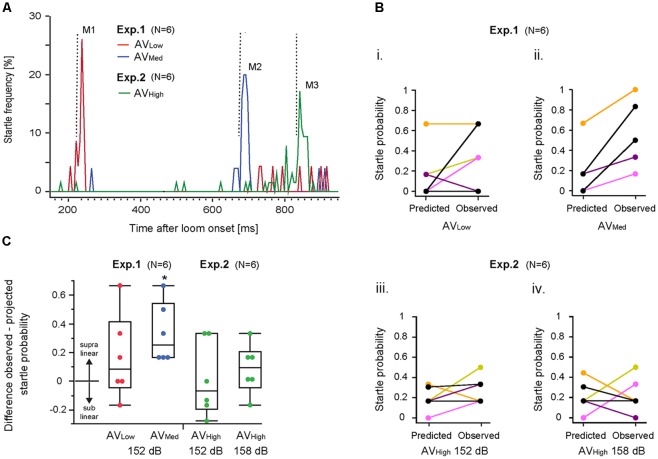
Multimodal integration in the M-cell startle system. **(A)** Line graph illustrates startle frequency plotted against visual loom duration for all three audio-visual (AV) stimulus paradigms. M1, M2, M3 represent distinct frequency modes for AV_Low_ and AV_Med_ or AV_High_ trials, respectively. Vertical dotted lines indicate onset of the auditory stimulus. **(B)** Graph illustrates observed and predicted individual startle escape probability for AV_Low_ and AV_Med_ stimulus paradigms using an auditory stimulus of 152 dB re 1 μPa in water **(i,ii)** and the AV_High_ paradigms involving auditory stimuli of 152 dB re 1 μPa in water (**iii**) and 158 dB re 1 μPa in water **(iv)**. Predicted startle escape probabilities were based on linear summation of response probabilities evoked in unimodal visual only and auditory only stimulus trials. Note: fish overlap in **(Bii)**. **(C)** Box plots of differences between observed minus predicted startle escape response probabilities for the indicated AV stimulus paradigm (*p = 0.043, one-sample *t*-test with a test value of 0, i.e., linear summation; *N* = 6).

We next analyzed startle rates for the different multimodal stimulus paradigms (i.e., AV_Low_, AV_Med_ and AV_High_) by graphing the frequency of responses over the duration of the loom (Figure 2A). Results show three response modes (Figure 2A: M1, M2, M3) within a time window typical for auditory responses (Figure 2A dotted lines and 1C) suggesting that they are due to a multimodal integration process that enhances responsiveness.

IEP predicts that multimodal integration disproportionately enhances responsiveness more for weaker than for stronger unimodal stimuli combinations (Meredith and Stein, 1986; Holmes, 2009). Accordingly, we compared the observed changes in startle probabilities in multimodal stimulus paradigms with those predicted by a linear summation of the unimodal auditory and visual startle probabilities (see “Materials and Methods” section for details). Visual only startle probabilities for the multimodal response modes (Figure 2A; M1, M2, M3) were derived from those occurring within 30 ms of a prospective auditory stimulus (arrows Figure 1D).

Results showed higher than predicted startle probabilities for individual fish for the AV_Med_ paradigm (Figure 2Bii; *M*_pred_ = 0.19 ± 0.25 vs. *M*_obser_ = 0.53 ± 0.32, Cohen’s *d* = 1.18). In contrast, responsiveness for the AV_Low_ paradigm (M_pred_ = 0.16 ± 0.25 vs. M_obser_ = 0.33 ± 0.29; Cohen’s *d* = 0.63) and the two AV_High_ paradigms (152 dB: M_pred_ = 0.21 ± 0.15 vs. M_obser_ = 0.22 ± 0.17, Cohen’s *d* = 0.06; 158 dB: M_pred_ = 0.19 ± 0.12 vs. M_obser_ = 0.28 ± 0.14 Cohen’s *d* = 0.69) was variable or even less than predicted for some fish (Figures 2Bi,iii,iv). Comparing the evoked changes for a given AV stimulus paradigm to a hypothetical value of zero (i.e., to a linear summation; two-tailed, single sample *t*-test) revealed a supralinear increase in startle probability for the AV_Med_ paradigm (Figure 2C; *p* = 0.0118; *p* = 0.04 after Benjamini-Hochberg correction). No significant differences to a linear summation of startle probabilities was found for AV_low_ (*p* = 0.23), AV_high_ 152 dB (*p* = 0.90), and AV_high_158 dB (*p* = 27; Figure 2C).

## Discussion

Here, we asked if the IEP (Meredith and Stein, 1986) applies for downstream sensorimotor neurons that directly initiate behavior such as the M-cells. Our findings largely support this notion. Specifically, we observed startle rates consistent with a linear integration of highly effective stimuli, but a supralinear multimodal integration to stimuli of reduced effectiveness (AV_med_), i.e., an inverse relationship between the individual effectiveness of two stimuli and their combined effectiveness. However, the AV_low_ paradigm did not produce the largest enhancement. Such a discrepancy to IEP might be due to stimulus floor effects (Holmes, 2009), and has been previously observed in for multimodal integration in the auditory cortex of primates (Lakatos et al., 2007).

Is the M-cell indeed the site of multimodal integration? Indeed, M-cell recordings in African cichlid fish and zebrafish revealed that a preceding light flash enhances auditory evoked synaptic currents, startle responsiveness, and directionality (Page and Sutterlin, 1970; Canfield, 2003, 2006; Mu et al., 2012). Importantly, chronic recordings in free-swimming goldfish and imaging in zebrafish showed visual loom stimuli and acoustic stimuli both trigger M-cell APs and initiate startle (Zottoli, 1977; Preuss et al., 2006; Weiss et al., 2006; Dunn et al., 2016). The presumed role of the M-cell is to initiate early parts of startle directly and/or to control threshold in segmental M-cell homologs, which are part of the brainstem escape network that produces later stages of the startle escape behavior (Liu and Fetcho, 1999; Gahtan et al., 2002; Kohashi and Oda, 2008; Nakayama and Oda, 2014; Neki et al., 2014). In other words, the M-cell is the first reticulospinal neuron active during a startle escape, or C-start, and the final common path for startle decisions (Zottoli, 1977; Fetcho, 1991; Weiss et al., 2006).

M-cell *in vivo* recordings showed that back propagating visual and auditory postsynaptic synaptic potentials (PSPs) interact at the dendritic level (Medan et al., 2018). Also, M-cell dendrites possess membrane non-linearities that enhance the effectiveness of such PSPs (Faber and Korn, 1986; Medan and Preuss, 2014). Both these properties likely contribute to the multimodal integration observed here. The latter notion however, does not exclude multimodal tectal neurons providing also critical input to the M-cell (Hiramoto and Cline, 2009; Truszkowski et al., 2017). Moreover, startle (i.e., M-cell) threshold is tightly controlled by at least two independent feedforward inhibition systems, which further influence sensory processing and multimodal integration (Preuss et al., 2006; Medan and Preuss, 2014; Medan et al., 2018). Together these findings suggest that a single neuron such as the M-cell can provide a neural correlate for the IEP phenomenon. In mammals, evidence for IEP in individual neurons derives from, e.g., recordings in cerebellar granule cells and superior colliculus neurons showing supralinear summation in spike rates during simultaneous auditory and visual stimulation (Ishikawa et al., 2015; Miller et al., 2015).

We used a stimulus combination that conceptually mimicked a diving bird breaking the water surface (Medan and Preuss, 2014). Thus, it is not surprising that all multimodal stimulus combinations enhanced startle escape responsiveness when compared to unimodal stimulus conditions. Functionally, such an enhancement might be particularly important when the salience of the individual stimuli is still low vs. a situation where stimuli are already highly salient (Holmes and Spence, 2005; ten Oever et al., 2016).

## Data Availability

All datasets generated for this study are included in the manuscript.

## Author Contributions

CM collected and analyzed data and wrote the manuscript. TP designed the study and revised the manuscript.

## Conflict of Interest Statement

The authors declare that the research was conducted in the absence of any commercial or financial relationships that could be construed as a potential conflict of interest.

## Footnotes

^1^http://research.hunter.cuny.edu/IACUC.htm
